# Contribution of Infant Rhinovirus Bronchiolitis to Hospital Bed and Ventilation Use

**DOI:** 10.1001/jamanetworkopen.2023.55033

**Published:** 2024-02-07

**Authors:** Côme Horvat, Jean-Sebastien Casalegno, Elsa Masson, Clémence Benveniste, Julie Haesebaert, John Paget, Dominique Ploin

**Affiliations:** 1 Hôpital Femme Mère Enfant, Service de Réanimation Pédiatrique et d’Accueil des Urgences, Hospices Civils de Lyon, Bron, France; 2 Hôpital de la Croix-Rousse, Centre de Biologie Nord, Institut des Agents Infectieux, Laboratoire de Virologie, Hospices Civils de Lyon, Lyon, France; 3 Research on Healthcare Performance, Institut National de la Santé et de la Recherche Médicale U1290, Université Claude Bernard Lyon 1, Hospices Civils de Lyon, Pôle Santé Publique, Service Recherche et Epidémiologie Cliniques, Lyon, France; 4 Nivel, Netherlands Institute for Health Services Research, Utrecht, the Netherlands

## Abstract

This cohort study compares the use of hospital resources related to human rhinovirus and respiratory syncytial virus infections among infants during 3 consecutive seasons before nirsevimab implementation.

## Introduction

Bronchiolitis is the main cause of infant hospitalization and ventilation worldwide. The most common pathogen is respiratory syncytial virus (RSV), which is intensively explored; other viruses, such as human rhinovirus (HRV), the second most common in bronchiolitis, are less documented.^1,2^ Studies on monoclonal antibodies, such as nirsevimab, and maternal vaccination^3^ reported promising results regarding RSV control, which could reduce RSV-related hospitalizations by more than 80%. However, these results may be impaired by other viruses; thus, we compared HRV- and RSV-related use of hospital resources during 3 consecutive seasons before nirsevimab implementation.

## Methods

We analyzed a historical cohort of infants hospitalized in a tertiary hospital in Lyon, France (86 000 visits/y to pediatric emergency units; 1.4 million–inhabitant catchment area), with laboratory-confirmed RSV or HRV infections (systematic multiplex reverse transcriptase-polymerase chain reaction for all infants with respiratory symptoms) during 3 seasons (July 1 to June 30 of 2019 to 2020, 2020 to 2021, and 2021 to 2022). A team of investigators reviewed infants’ medical records to confirm bronchiolitis diagnosis.

The HRV-related burden was expressed as a percentage of the RSV-related burden for the number of days of hospitalization or ventilation use. The emergency bed capacity, which refers to the beds available in the emergency and pediatric intensive care units, and the emergency ventilation capacity, which is restricted to the beds available in the pediatric intensive care unit, were displayed as 100% and 50% lines in the temporal distribution histograms. Coinfections of RSV and HRV were equally attributed with a 0.5 weighting. The study was approved by Comité Scientifique et Ethique du CHU des Hospices Civils de Lyon, and an information letter was sent to the parents with the option to refuse the use of the individual data. We followed the STROBE reporting guideline.

## Results

**Figure 1.**
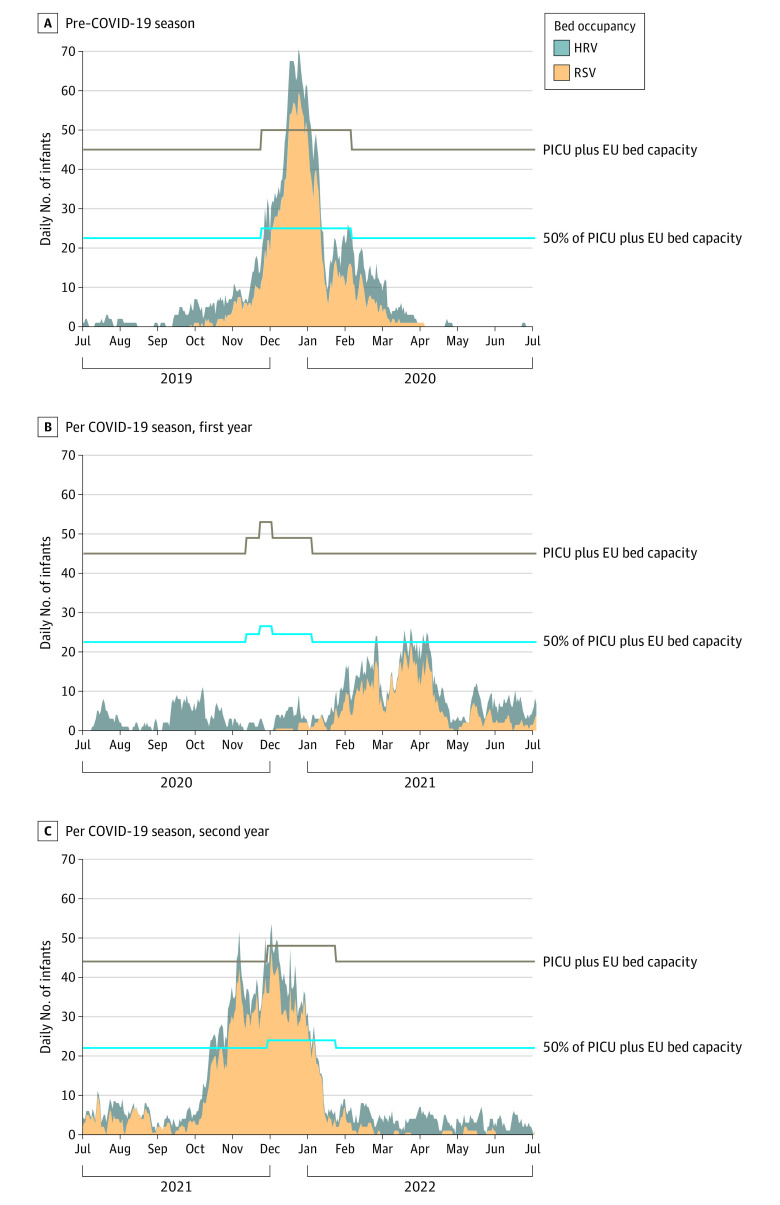
Daily Use of Emergency Beds for Respiratory Syncytial Virus (RSV) and Human Rhinovirus (HRV) Among Infants, 2019 to 2022 Each season begins on July 1 and ends on June 30. EU indicates emergency unit; PICU, pediatric intensive care unit.

**Figure 2.**
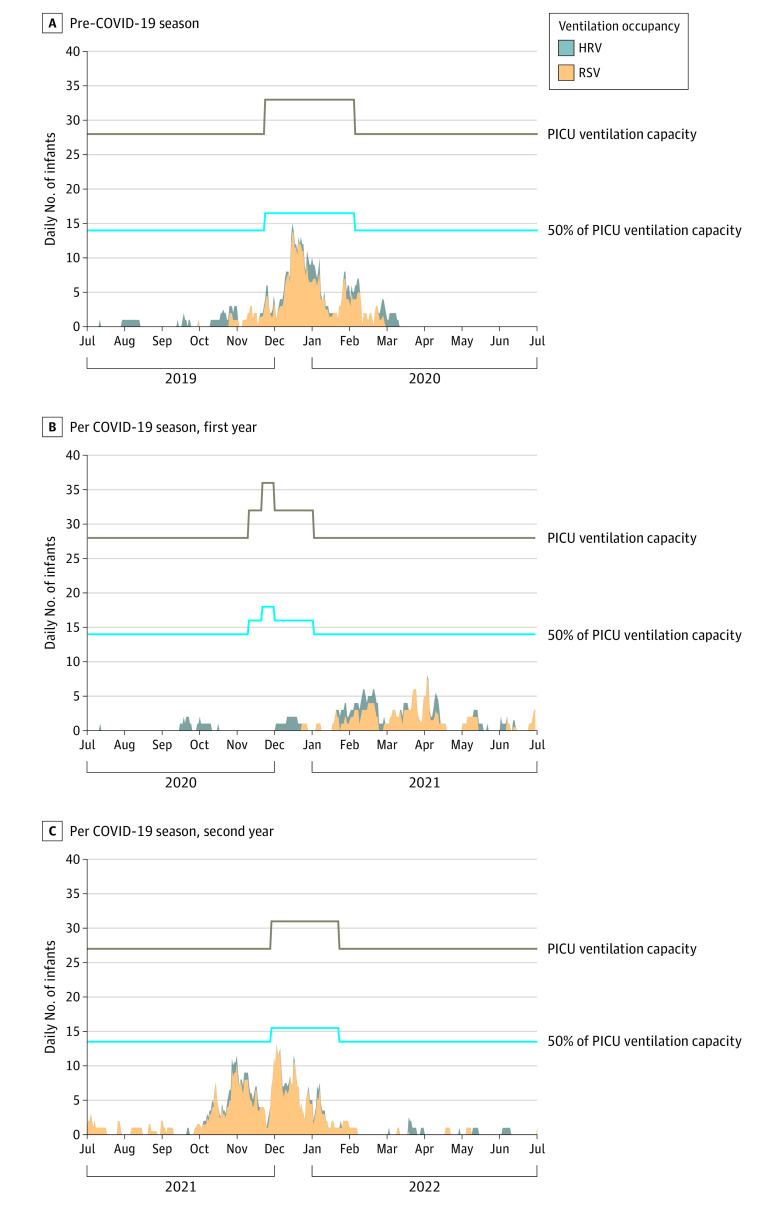
Daily Use of Emergency Ventilation Beds for Respiratory Syncytial Virus (RSV) and Human Rhinovirus (HRV) Among Infants, 2019 to 2022 Each season begins on July 1 and ends on June 30. PICU indicates pediatric intensive care unit.

From July 1, 2019, to June 30, 2022, a total of 1122 RSV (597 [53%] boys and 525 girls [47%]) and 766 HRV (447 [58%] boys and 319 [42%] girls) infections were identified (ie, admission rate due to HRV was 68% of the total for RSV). The hospitalization ratio of HRV to RSV days was 51% (3608 of 7017 infant-days), and for days of ventilation, 27% (392 of 1426 infant-days). The temporal distribution showed that HRV-related burden was spread across the year, while RSV-related burden was seasonal (Figure 1 and Figure 2). More than 50% of the emergency bed capacity was used during 47 (2019-2020), 9 (2020-2021), and 80 (2020-2021) days. More than 100% of the emergency bed capacity was used during 20 (2019-2020) and 9 (2021-2022) days. The use of emergency ventilation capacity never exceeded 50%.

## Discussion

To our knowledge, this cohort study is the first to show that the magnitude of HRV burden was up to two-thirds that of RSV-related hospital admissions, half of RSV-related days of hospitalization, and one-quarter of RSV-related days of ventilation; both burdens are cumulative. We did not explore every cause of the hospitalization rate’s fluctuation but aimed to express the relative part of HRV in the burden of respiratory infections. Our findings suggest that the promised 86.5% individual efficacy of nirsevimab may not translate to an 86.5% reduction in bronchiolitis burden. As nirsevimab will certainly reduce the burden due to bronchiolitis and therefore reduce care cancellation, hospital transfers, and other substantial quality of care impairments, our findings do not undermine its implementation; however, they draw attention to challenges regarding bronchiolitis management, especially non-RSV infections. To protect hospital and nonhospital pediatric settings from the devastating effects of multipathogen winter epidemics, efforts must be made to include HRV in the development of multipathogen vaccines^4^ and to continue to optimize patient management by developing tailored protocols for home enteral nutrition and home oxygen therapy for infants with moderately severe bronchiolitis.^5^ Until then, adherence to universal preventive hygiene measures remains particularly critical.^6^

## Notes

Supplement.
Data Sharing Statement
