# Supplementary material for: Contribution of Infant Rhinovirus Bronchiolitis to Hospital Bed and Ventilation Use
Source: JAMA Netw Open. 2024 Feb 7;7(2):e2355033. doi: 10.1001/jamanetworkopen.2023.55033 (PMC10851092; doi:10.1001/jamanetworkopen.2023.55033)
Supplement: Supplement. — Data Sharing Statement [file jamanetwopen-e2355033-s001.pdf]

Data Sharing Statement

Horvat. Infant Rhinovirus Bronchiolitis as a Major Contributor of Hospital Bed and Ventilation Use. *JAMA Netw Open*. Published February 07, 2024.
doi:10.1001/jamanetworkopen.2023.55033

Data
Data available: Yes
Data types: Deidentified participant data
How to access data: Data will be available upon reasonable request to the corresponding author
When available: Beginning date: 06-27-2024

Supporting Documents
Document types: None

Additional Information
Who can access the data: Researchers whose proposed use of the data has been approved
Types of analyses: The data will be made available for further epidemiological studies
Mechanisms of data availability: The data will be made available with investigator support, after approval of a proposal.
